# Supplementary material for: Comparative Assessment of the Anti-Helicobacter pylori Activity and Gastroprotective Effects of Three Herbal Formulas for Functional Dyspepsia In Vitro
Source: Cells. 2024 May 24;13(11):901. doi: 10.3390/cells13110901 (PMC11172274; doi:10.3390/cells13110901)
Supplement: Supplementary file 1 [file cells-13-00901-s001.zip › cells-3001962-supplementary.pdf]

Supplementary data

Table S1. Primer information for qPCR.

Names		Sequences	Primer Length (bp)
GAPDH	F	5'-TGGGCTACACTGAGCACCAG-3'	51
	R	5'-GGGTGTCGCTGTTGAAGTCA-3'	
TLR-4	F	5'-GGTGGAAGTTGAACGAATGG-3'	186
	R	5'-CCAGCAAGAAGCATCAGGTG-3'	
IL-6	F	5'-CCTTGGGTCCAGTTGCCTTCT-3'	234
	R	5'-CCAGTGCCTCTTTGCTGCTTTC-3'	
IL-8	F	5'-CTTTCAGAGACAGCAGAG-3'	183
	R	5'-CTAAGTTCTTTAGCACTCC-3'	
IL-16	F	5'-AGTTCCCTTCAGTCTGGTCAG-3'	150
	R	5'-AGCACCTTCCTCCTTGTGTAAG-3'	
TNF-α	F	5′-CAAGCCTGTAGCCCATGTTGTAGC-3′	432
	R	5′-ATCCCAAAGTAGACCTGCCCAGAC-3′	
COX-2	F	5'-AGTCCCTGAGCATCTACGGT-3'	352
	R	5'-AAAGGTGTCAGGCAGAAGGG-3'	
Occludin	F	5'-GTCCAATATTTTGTGGGACAAGG-3'	99
	R	5'-GGCACGTCCTGTGTGCCT-3'	
Claudin	F	5'-CGCGAGAAGAAGTACACGG-3'	107
	R	5'-CCTTAGACGTAGTCCTTGCGG-3'	

Abbreviations

GAPDH: glyceraldehyde-3-phosphate dehydrogenase; TLR: Toll-like receptor; IL: interleukin; TNF-α: tumor necrosis factor-alpha; COX: cyclooxygenase; F: forward; R: reverse; bp: base pair.
